# Factors associated with chronic frequent emergency department utilization in a population with diabetes living in metropolitan areas: a population-based retrospective cohort study

**DOI:** 10.1186/s12913-017-2453-3

**Published:** 2017-08-04

**Authors:** Catherine Hudon, Josiane Courteau, Cynthia Krieg, Alain Vanasse

**Affiliations:** 1 Groupe de recherche PRIMUS, Centre de recherche du Centre hospitalier universitaire de Sherbrooke (CRCHUS), 3001 12e avenue nord, Sherbrooke, QC J1H 5N4 Canada; 2 Département de médecine de famille et de médecine d’urgence, Université de Sherbrooke, 3001 12e avenue nord, Sherbrooke, QC J1H 5N4 Canada

**Keywords:** Emergency department, Diabetes, Frequent use, Administrative data

## Abstract

**Background:**

A small proportion of patients utilizes a disproportionately large amount of emergency department (ED) resources. Being able to properly identify chronic frequent ED users, i.e. frequent ED users over a multiple-year period, would allow healthcare professionals to intervene before it occurs and, if possible, redirect these patients to more appropriate health services. The objective of this study was to explore the factors associated with chronic frequent ED utilization in a population with diabetes.

**Methods:**

A population-based retrospective cohort study using administrative data was conducted on 62,316 patients with diabetes living in metropolitan areas of Quebec (Canada), having visited an ED during 2006, and still alive in 31 December 2009. The dependant variable was being a chronic frequent ED user, defined as having at least 3 ED visits per year during three consecutive years (2007–2009). Independent variables, measured during 2006, included age, sex, neighbourhood deprivation, affiliation to a general practitioner, and number of physical and mental health comorbidities. Logistic regression and tree-based method were used to identify factors associated with chronic frequent ED use.

**Results:**

A total of 2.6% of the cohort (patients with diabetes and at least one ED visit in 2006) was identified as chronic frequent ED users. These patients accounted for 16% of all ED visits made by the cohort during follow-up. The cumulative effect of a high illness burden combined with mental health disorders was associated with an increased risk of chronic frequent ED use.

**Conclusions:**

Interventions must target the population at higher risk of becoming chronic frequent ED users and should be designed to manage the complex interaction between high illness burden and mental health.

## Background

Emergency department (ED) overcrowding has become a critical issue for many hospitals [1] and it is well acknowledged that a small proportion of patients uses a disproportionately large amount of ED resources [1, 2]. Many studies discussed the concept of frequent users [3–9] but few of them used a standard definition. However, frequently used definitions are at least three or four ED visits during a 1-year period [1, 10–14]. The use of ED services by frequent users can often be perceived as inappropriate and non-urgent [15, 16], resulting in uncoordinated and less effective care as compared to what these patients would receive in primary care [17, 18]. This situation generates substantial costs to the health care system [19, 20], it decreases ED efficiency [2], and contributes to ED overcrowding [21, 22].

Being able to properly identify chronic frequent ED users (CFUs), i.e. frequent ED users over a multiple-year period, would allow healthcare professionals to intervene before it occurs and, if possible, redirect these patients to more appropriate health services [23]. A recent scoping review on individual predictors of frequent ED use and CFU [24] found that, in general, frequent ED users over 1 year had a low socioeconomic status, high levels of health care use (other than the ED), and suffered from multiple physical and mental health conditions. To date, however, very few studies have focussed on CFU.

Patients living with diabetes are known to be high health care users [25, 26]. In a study conducted on patients with cardiovascular risk factors (including diabetes), about 5% used near 50% of all ED visits made by that population [10]. In another study conducted specifically on patients with diabetes living in a metropolitan area (Montreal, Canada) [27], patients living in materially or socially deprived neighbourhoods were more likely to frequently visit EDs.

To our knowledge, no study has analysed CFU in the population with diabetes. Furthermore, a better understanding of factors associated with CFU is critical in order to improve care, reduce their ED visits and associated costs by direct effective interventions. The objective of this study was thus to explore the factors associated with CFU, i.e. frequent ED use for three consecutive years, in a population with all forms of diabetes.

## Methods

### Design and data sources

This is a population-based retrospective cohort study. Patient data were obtained from the provincial health insurance board (*Régie de l’assurance maladie du Québec*: RAMQ), which provides universal health insurance to Quebec residents, including coverage for physician and hospital services. The RAMQ owns and manages administrative health registers including hospital discharges (MED-ECHO), patients’ demographic information, and physicians’ reimbursement claims for health care (including hospital inpatients and outpatients, emergency and private clinics). The MED-ECHO registry contains information on dates of hospitalizations, length of stay, the main and secondary diagnoses (ICD-9 before 2006, ICD-10 thereafter). The RAMQ demographic database provides information on patients’ age, gender, and date of death. The physician reimbursement claims register provides the date of service and the diagnosis (ICD-9) specific to the medical visit. Using a unique encrypted identifier, patient data from these registers were linked to provide information on demographic characteristics and medical information. In order to use neighbourhood information such as material and social deprivation as provided by the 2006 Census of population for dissemination areas (DA), each patient was spatially linked to one and only 1 DA using the postal code conversion file (PCCF) from Statistics Canada.

### Case definition of diabetes

A patient was considered living with diabetes (any form) if he/she had received a primary or secondary diagnosis of diabetes (ICD-9: 250; ICD-10: E10-E14) during a hospitalization or had at least three physician claims within 1 with a diagnosis of diabetes. This case definition have also been used elsewhere [14, 27].

### Studied population

The studied population included all individuals aged ≥30 years with any form of diabetes (according to the case definition above) between January 1999 and December 2006, alive at the end of 2006, having visited an ED at least once during 2006. Since rural residents have often limited access to EDs (in terms of proximity) and the pattern of use of EDs may differ between urban and rural areas [28], we restricted the study population to patients living in one of the six metropolitan areas of the province of Quebec, Canada (Montréal, Québec, Gatineau, Sherbrooke, Trois-Rivières, Saguenay). All patients that died during the 3 years follow-up period (2007–2009) were excluded.

### Variables

The following binary dependent variable was defined: being a CFU (≥3 visits per year for three consecutive years: 2007, 2008 and 2009). ED visits made on consecutive days were considered referring to the same ED episode and were counted only once. The choice of using a threshold of 3 ED visits to define frequent users was based on the distribution of ED visits: 12.9% of patients made 57.0% of all ED visits in 2007.

We accounted for the following independent variables measured before follow-up: sex, age, being affiliated to a general practitioner (GP), having visited in 2006 an endocrinologist, an ophthalmologist or an internist, living in a materially or socially deprived neighbourhood (corresponding to the 2 most deprived quintiles), having been hospitalized for any cause in 2006, the D’Hoore adaptation of the Charlson comorbidity index [29] (excluding diabetes), the presence of specific physical comorbidities (complications of diabetes, high blood pressure, dyslipidemia, injury, chronic obstructive pulmonary disease (COPD), cardiovascular disease (CVD), cancer, renal disease, liver disease, connective tissue disease, ulcer disease), and the presence of mental health disorders (substance abuse, dementia, other mental health disorder). The physical and mental health comorbidities were calculated using the diagnoses reported in MED-ECHO and in the physicians’ claims register between January and December 2006 (Table 1). Most of the selected comorbidities are listed in the comorbidity index, and some are related to the diabetes condition (complications of diabetes, high blood pressure, dyslipidemia). To determine if a patient was affiliated to a GP, we considered all ambulatory visits to GPs (excluding at EDs) during the 2-year period (2005–2006) before follow-up. A patient was considered affiliated to a GP if at least 75% of all these visits were made to the same GP. If a patient had only one visit to a GP during that period, he/she was considered affiliated to a GP [30]. Neighbourhoods were considered materially or socially deprived if they belonged to the two most deprived population quintiles (4th or 5th quintiles) according to the Pampalon deprivation index [31].

**Table 1:** Comorbidities and their associated classification codes*

Comorbidity	ICD-9	ICD-10
Mental health comorbidities		
Any mental health dis.	290–319	F00-F99
Substance abuse	291, 292, 303, 304, 305	F10-F19, F55
Dementia	290, 291, 294	F00-F06, F09, F10
Other mental health dis.	290–319 except 290–292, 294, 303, 304, 305	F00-F99 except F00-F06, F09 -F19, F55
Physical comorbidities		
Complication of diabetes	250.1–250.9	E10-E14 not E10.9
High blood pressure	401–405	I10-I15
Dyslipidemia	272	E78, E75.2–3–5-6, E77.0–9, E88.1–2, H026
Injury	800–999	S00-T99
Cardiovascular disease (CVD):		
Myocardial infarction	410, 411	I20.0, I21, I22, I24, I51.3
Congestive heart failure	298, 402, 428	I09.0, I11, I50
Peripheral vascular disease	440–447	I70-I77
Cerebrovascular disease	430–435	G45.0-G45.2, G45.4, G45.8, G45.9, I60-I62
Chronic obstructive pulmonary disease (COPD)	491–493	J41-J45
Connective tissue disease	710, 714, 725	M05, M06, M08, M09, M12, M32-M36, L871
Ulcer disease	531–534	K25-K28
Liver disease	571, 573	K70, K71, K73-K77
	070, 570, 572	B15-B19, K72
Renal disease	403–404, 580–586	I12-I13, N00, N01, N03-N05, N07, N08, N14, N17-N19, N150, N16.3, N29.0
Cancer:		
Any tumor	140–195	C00-C76
Leukemia	204–208	C91-C95
Lymphoma	200–203	C81-C86, C88, C90, C96
Metastatic solid tumor	196–199	C77-C80, C97

*The majority of the comorbidities are conditions listed in the D’Hoore adaptation of the Charlson comorbidity index

### Statistical analysis

Multiple logistic regression was used to model the odds of being a CFU including all independent variables defined above, using SAS software Version 9.4. We also performed tree-based analyses using the RTREE program [32, 33] by sex and age group (30–54; ≥55). Tree-based analysis is a nonparametric method of recursive partitioning allowing identification of hierarchically organized risk factors for a dichotomous outcome. This approach has the advantage of taking into account interactions between independent variables and forming homogeneous profiles of populations according to their risk of outcome. The relevance of this method to investigate health issues using RAMQ data has been demonstrated by Vanasse et al. [34, 35].

## Results

**Fig. 1:**
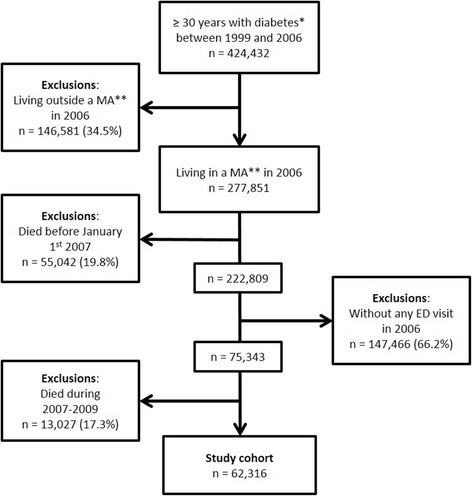
Study cohort flow diagram. * A patient was considered living with diabetes if he/she received a diagnosis of diabetes (ICD-9: 250; ICD-10: E10-E14) during a hospitalization or at least three physician claims within 1 year with an identical diagnosis. ** MA: Metropolitan area

The study cohort included 62,316 patients with diabetes (Fig. 1), among which 8031 (12.9%) visited an ED at least 3 times during 2007, 2961 (4.8%) visited an ED at least 3 times per year for two consecutive years (2007–2008), and 1606 (2.6%) visited an ED at least 3 times per year for three consecutive years (2007–2009). Hence, only 20% of frequent ED users in 2007 became CFUs. These latter patients represent the CFU group. As compared to non-CFUs, they were more often women, younger, lived in more deprived neighbourhoods, have been previously hospitalized in a higher proportion, and had more physical and mental health comorbidities (Table 2).

**Table 2:** Characteristics of the study cohort living with diabetes and factors associated with the risk of being a chronic frequent ED user (CFU)

Characteristics	Total, n (%)	Non-CFUs, n (%)	CFUs, n (%)	Crude OR (95% CI)	Age-sex adj. OR* (95% CI)	Fully adj. OR^**†**^ (95% CI)
Total, n (%)	62,316 (100)	60,710 (97.4)	1606 (2.6)	−	−	−
Sex						
Male	30,938 (49.6)	30,457 (50.2)	685 (42.6)	Ref	Ref	Ref
Female	31,378 (50.4)	30,253 (49.8)	921 (57.4)	1.34 (1.21–1.48)	1.34 (1.21–1.48)	1.27 (1.14–1.41)
Age group						
30–54 years	14,801 (22.6)	13,618 (22.4)	463 (28.8)	Ref	Ref	Ref
55–64 years	14,949 (24.0)	14,646 (24.1)	303 (18.9)	0.61 (0.52–0.70)	0.62 (0.53–0.71)	0.62 (0.53–0.72)
65–74 years	16,214 (26.0)	15,803 (26.0)	411 (25.6)	0.76 (0.67–0.88)	0.76 (0.67–0.87)	0.76 (0.66–0.88)
75 + years	17,072 (27.4)	16,643 (27.4)	429 (26.7)	0.76 (0.66–0.87)	0.73 (0.64–0.84)	0.73 (0.62–0.85)
Material deprivation	22,239 (35.7)	21,551 (35.5)	688 (42.8)	1.36 (1.23–1.51)	1.34 (1.21–1.48)	1.28 (1.16–1.42)
Social deprivation	32,054 (51.4)	31,089 (51.2)	965 (60.1)	1.43 (1.30–1.59)	1.42 (1.28–1.57)	1.28 (1.15–1.42)
Affiliation to a GP	37,227 (59.7)	36,383 (59.9)	844 (52.6)	0.74 (0.67–0.82)	0.77 (0.69–0.85)	0.81 (0.73–0.89)
Visit to an endocrinologist	11,941 (19.2)	11,605 (19.1)	336 (20.9)	1.12 (0.99–1.26)	1.09 (0.96–1.23)	1.10 (0.97–1.25)
Visit to an ophthalmologist	22,974 (36.9)	22,404 (36.9)	570 (35.5)	0.94 (0.85–1.04)	0.96 (0.87–1.07)	1.00 (0.90–1.12)
Visit to an internist	10,480 (16.8)	10,154 (16.7)	326 (20.3)	1.27 (1.12–1.44)	1.29 (1.14–1.45)	1.07 (0.94–1.21)
Substance abuse	2665 (4.3)	2457 (4.1)	208 (13.0)	3.53 (3.03–4.10)	3.60 (3.09–4.19)	2.04 (1.71–2.43)
Dementia	4011 (6.4)	3849 (6.3)	162 (10.1)	1.66 (1.40–1.96)	1.74 (1.47–2.07)	0.61 (0.50–0.75)
Other mental disorder	13,967 (22.4)	13,305 (21.9)	662 (41.2)	2.50 (2.26–2.76)	2.41 (2.17–2.67)	1.91 (1.72–2.13)
Hospitalization in 2006	28,664 (46.0)	27,655 (45.6)	1009 (62.8)	2.02 (1.82–2.24)	2.10 (1.89–2.33)	1.03 (0.89–1.19)
Charlson Comorbidity index (CCI)						
0	32,716 (52.5)	32,226 (53.1)	490 (30.5)	Ref	Ref	Ref
1	10,959 (17.6)	10,601 (17.5)	358 (22.3)	2.22 (1.94–2.55)	2.39 (2.08–2.74)	1.56 (1.31–1.86)
2	7937 (12.7)	7694 (12.7)	243 (15.1)	2.08 (1.78–2.43)	2.38 (2.04–2.80)	1.77 (1.42–2.21)
3 +	10,704 (17.2)	10,189 (16.8)	515 (32.1)	3.32 (2.93–3.77)	3.96 (3.47–4.52)	2.21 (1.63–2.99)
Complications of diabetes	22,778 (36.6)	21,982 (36.2)	796 (49.6)	1.73 (1.57–1.91)	1.78 (1.61–1.97)	1.08 (0.96–1.23)
High blood pressure	27,639 (44.4)	26,791 (44.1)	848 (52.8)	1.42 (1.28–1.56)	1.51 (1.36–1.68)	0.99 (0.88–1.11)
Dyslipidemia	12,640 (20.3)	12,186 (20.1)	454 (28.3)	1.57 (1.40–1.75)	1.65 (1.48–1.84)	0.95 (0.84–1.08)
Injury	24,845 (39.9)	23,943 (39.4)	902 (56.2)	1.97 (1.78–2.17)	1.96 (1.78–2.17)	1.71 (1.54–1.89)
COPD	7657 (12.3)	7161 (11.8)	496 (30.9)	3.34 (3.00–3.72)	3.34 (3.00–3.73)	2.01 (1.75–2.30)
CVD	14,513 (23.3)	13,955 (23.0)	558 (34.7)	1.78 (1.61–1.98)	2.00 (1.80–2.23)	1.11 (0.95–1.28)
Cancer	7467 (12.0)	7247 (11.9)	220 (13.7)	1.17 (1.01–1.35)	1.24 (1.08–1.44)	0.78 (0.64–0.96)
Renal disease	7575 (12.2)	7231 (11.9)	344 (21.4)	2.02 (1.78–2.28)	2.18 (1.93–2.47)	1.08 (0.90–1.30)
Liver disease	2422 (3.9)	2282 (3.8)	140 (8.7)	2.44 (2.05–2.92)	2.47 (2.06–2.95)	1.27 (1.04–1.56)
Connective tissue dis	1298 (2.1)	1253 (2.1)	45 (2.8)	1.37 (1.01–1.85)	1.34 (0.99–1.82)	0.94 (0.69–1.29)
Ulcer disease	1041 (1.7)	989 (1.6)	52 (3.2)	2.02 (1.52–2.68)	2.11 (1.59–2.80)	1.27 (0.94–1.70)

^*^Adjusted only for age and sex. For the variables age and sex, the model is adjusted for the other variable

^†^Adjusted for all variables of Table 2

**Fig. 2:**
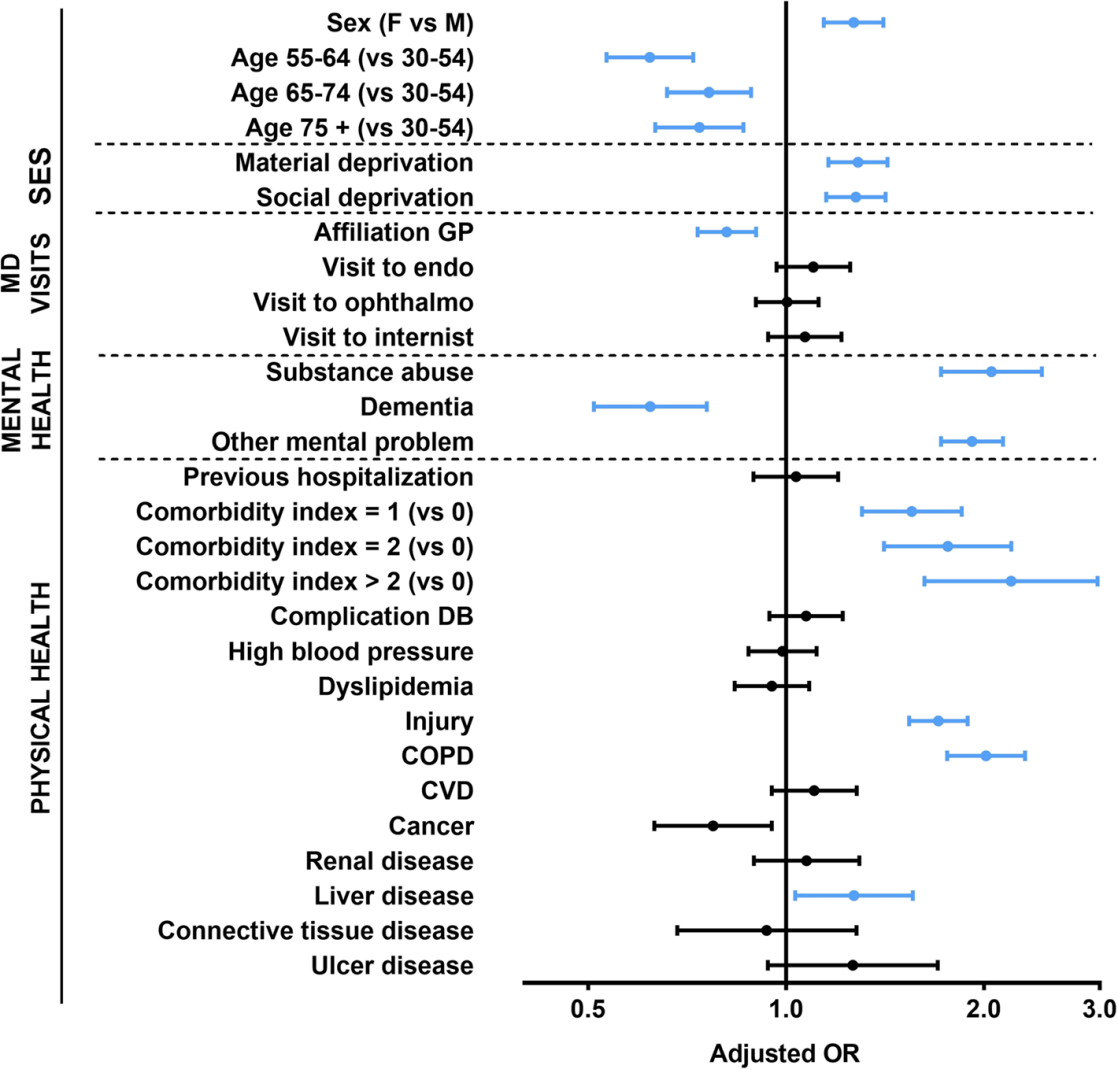
Risk of being a chronic frequent ED user (CFU) in a population with diabetes: multiple logistic regression analysis* (*n* = 62,316). *Adjusted for all independent variables (fully adjusted model)

The most important predictors (i.e. those with the highest odds ratios) of CFU were (Fig. 2, Table 2): having a higher comorbidity index, a substance abuse diagnosis, a mental health diagnosis, a diagnosis of COPD, and a diagnosis of injury. Being a woman, having a diagnosis of a liver disease, and living in deprived neighbourhoods were also associated with an increased risk of CFU, but to a lesser extent. Conversely, older age, having a diagnosis of dementia, and being affiliated to a GP were all associated with a reduced odds of CFU.

**Fig. 3:**
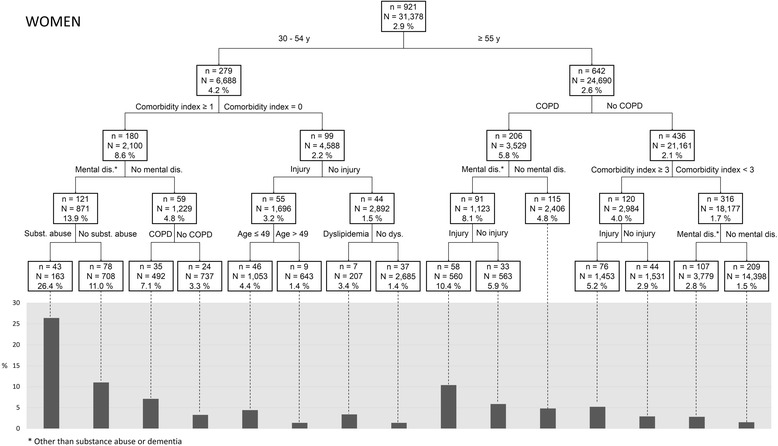
Risk profiles of being a chronic frequent ED user (CFU) in a women population with diabetes: tree-based approach*. *In each box, n represents the number of CFUs and N the total number of patients (CFUs and Non-CFUs). The percent represents the proportion of CFUs in the subgroup

**Fig. 4:**
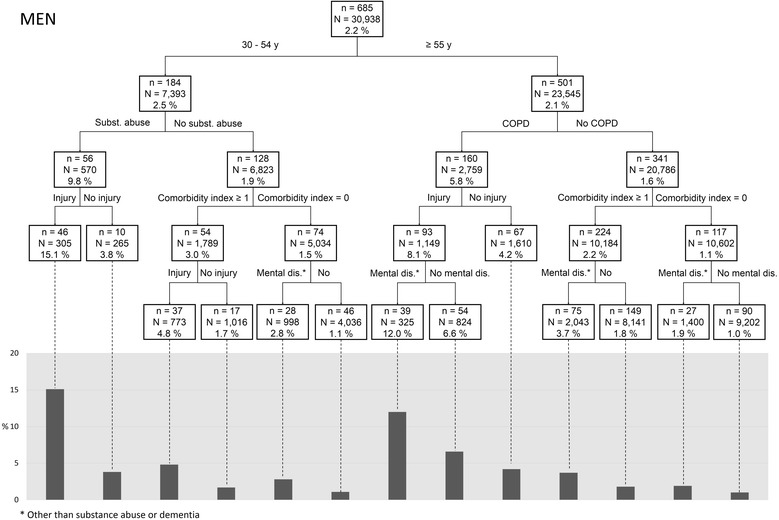
Risk profiles of being a chronic frequent ED user (CFU) in a men population with diabetes: tree-based approach*. *In each box, n represents the number of CFUs and N the total number of patients (CFUs and Non-CFUs). The percent represents the proportion of CFUs in the subgroup

Profiles generated by the tree-based analysis by sex and age group (Figs. 3 and 4) included the subgroup with the most important proportion of CFUs (26.4%), namely female patients aged 30–54 years with a nonzero comorbidity index and diagnosed during 2006 with a mental health disorder (other than dementia) and substance abuse. Predictors remain essentially the same between women and men and between younger and older patients, younger female patients being more at risk of being CFU than male patients.

## Discussion

This study is one of the few to evaluate predictive factors of CFU and the only one in a population with diabetes. A total of 2.6% of patients with diabetes were identified as CFUs, which means that they were frequent ED users (≥3 ED visits) for three consecutive years. In addition, these few patients cumulated a large proportion (16%) of all ED visits made by the cohort during 3 years (2007–2009).

The cumulative effect of a high illness burden (comorbidity index, COPD, injury) combined with mental health disorders (substance abuse, mental health disorder other than dementia) was associated with an increased risk of CFU. Moreover, being younger (30–54), a woman, and living in deprived neighbourhoods intensified that risk. Tree-based analyses provide additional information that may be helpful to clinicians by generating subgroups particularly at risk of being CFU. For example, 26.4% of women aged 30–54 years with a nonzero comorbidity index, a diagnosed mental health disorder and substance abuse were CFUs, whereas 15.1% of men aged 30–54 years diagnosed with substance abuse and injury were CFUs. Patients without comorbidities (physical and mental) had very low risk of being CFUs.

As reported in a recent scoping review [24], only four studies [36–39] examined specifically the factors associated with CFU. These factors included: previous ED utilization, having contact with psychiatric care, living alone and perceived loneliness, and having multiple chronic conditions including mental health disorders. Our study not only confirms that high illness burden (especially COPD and injury), and mental health disorders are associated with CFU, it provides specific subgroups particularly at risk of CFU, such as younger women with combined high illness and mental health burden.

Many frequent ED users present chronic conditions that should be cared for by primary ambulatory care: Ambulatory Care Sensitive Conditions (ACSC). ACSC are chronic conditions for which adequate ambulatory care can prevent deterioration or complications requiring visits to the ED or hospitalisations [40], such as asthma, COPD, diabetes, epilepsy, high blood pressure, heart failure and atherosclerotic cardiovascular disease [41].

Primary care should be organized in order to meet the needs of patients with a high illness burden and mental health comorbidity. In fact, as much as 10% of CFUs had COPD, injury and a mental health disorder and almost 40% had two of these illnesses or disorders, as opposed to less than 2% and 15%, respectively, of non-CFUs (data not shown). The combination of these disorders are not infrequent in a population already living with diabetes. Targeting especially these complex patients may have a positive impact on their health needs and on the healthcare delivery. An interdisciplinary approach with health professionals, including mental health and social services is essential. Considering the complexity of these CFUs, case management is often suggested to promote better integration of health and social services [42, 43].

Another implication of the findings is the need to encourage policy makers to prioritize efforts to reduce the factors contributing to deprived neighbourhoods. These include inadequate income for individuals and families as well as insufficient affordable housing. Reducing barriers that inhibit access to mental health treatment is another important avenue.

### Strengths and limitations

Strengths are related to the large number of patients included in the cohort (*n* = 62,316) and the fact that the study reflects a real-world situation. Also, a tree-based approach was used to describe specific profiles of patients with diabetes according to their risk of being CFU. The main limitation is related to the use of administrative databases. First, socioeconomic information was not available at the individual level in administrative data, so we used a socioeconomic proxy at the neighbourhood-level, which may lead to some ecological bias [44]. Since this study was performed on a specific subpopulation (with diabetes) living in metropolitan areas, these results may not be generalizable to the general population limiting its external validity. Finally, although the algorithm used to identify diabetes cases has not been explicitly validated and differs from the National Diabetes Surveillance System definition, we can expect that the algorithm used for this study will have a low sensitivity but a very high specificity.

## Conclusion

In conclusion, CFUs are infrequent (2.6%) among patients with diabetes, but they cumulated 16% of all ED visits made by the study cohort over a 3-year period. Interventions must target the population at higher risk of becoming CFU and should be designed to manage the complex interaction between diabetes, other chronic conditions and mental health disorders.

## Abbreviations

CFU: Chronic frequent ED user
COPD: Chronic obstructive pulmonary disease
CVD: Cardiovascular disease
DA: Dissemination area
ED: Emergency department
GP: General practitioner
ICD: International classification of diseases
PCCF: Postal code conversion file
RAMQ: Régie de l’assurance maladie du Québec
